# Establishing a Net-Zero Emissions Kidney Care Center: A Model Proposal for Taiwan

**DOI:** 10.2196/73942

**Published:** 2025-09-22

**Authors:** Mei-Yi Wu, Wei-Cheng Lo, Min-Kuang Tsai, Yuan-Leng Lin, Yih-Giun Cherng, Ming-Che Lee, Mai-Szu Wu

**Affiliations:** 1 Division of Nephrology, Department of Internal Medicine Shuang Ho Hospital Taipei Medical University New Taipei Taiwan; 2 Division of Nephrology, Department of Internal Medicine School of Medicine, College of Medicine Taipei Medical University Taipei Taiwan; 3 Taipei Medical University Research Center of Urology and Kidney Taipei Medical University Taipei Taiwan; 4 Master Program in Applied Epidemiology College of Public Health Taipei Medical University Taipei Taiwan; 5 School of Public Health, College of Public Health Taipei Medical University Taipei Taiwan; 6 Department of Anesthesiology School of Medicine, College of Medicine Taipei Medical University Taipei Taiwan; 7 Department of Anesthesiology Shuang Ho Hospital Taipei Medical University New Taipei Taiwan; 8 Department of Surgery School of Medicine, College of Medicine Taipei Medical University Taipei Taiwan; 9 Division of General Surgery, Department of Surgery Shuang Ho Hospital Taipei Medical University New Taipei Taiwan

**Keywords:** net-zero carbon emissions, green nephrology, sustainable kidney care, digital transformation, circular economy, disease prevention

## Abstract

Green nephrology has emerged as a crucial strategy to address the health care sector’s role in the climate crisis, particularly due to the high carbon intensity of dialysis-related services. Aligned with global net-zero commitments, sustainable kidney care can reduce environmental impact while maintaining high standards of patient care. This viewpoint paper proposes a net-zero carbon emissions kidney care center model to address global climate change challenges and advance health care sustainability goals. Based on the United Nations Sustainable Development Goals, we developed a 4D framework: digital transformation, low-carbon health care, circular economy, and preventive medicine. The digital transformation dimension features a precision kidney health system integrating acute and chronic kidney injury digital care models. The low-carbon health care dimension focuses on increasing the rates of kidney transplantation and choosing optimal dialysis modality. The circular economy dimension involves dialysis wastewater recycling, repurposing of medical materials, and integration of renewable energy into facility operations. The preventive medicine dimension incorporates telehealth education, behavioral interventions, and health inequality improvements. This net-zero carbon emissions kidney care model represents an environmental, social, and governance approach to ensuring implementation and continual improvement. It also provides actionable steps for implementing sustainable kidney care and serves as a reference model for net-zero emissions health care systems.

## Introduction

### Green Nephrology

Green nephrology is crucial to global efforts to achieve the United Nations’ objective of net-zero carbon emissions by 2050 [1]. This shift toward environmentally sustainable kidney care aims to reduce nephrology services’ carbon footprint and ecological influence without compromising patient outcomes. Nephrology, especially hemodialysis, is particularly carbon-intensive due to the use of energy, water, and consumables and the repetitive nature of treatments [2-4].

The Paris Agreement, adopted at the 21st Conference of the Parties of the United Nations’ Framework Convention on Climate Change in 2015, marked a key milestone in global climate action [5]. This agreement commits signatory countries to implement decarbonization policies to limit global temperature increases to <2 °C, optimally within 1.5 °C, by the end of the 21st century. The 26th Conference of the Parties, held in Glasgow, Scotland, in 2021, extended the objective of mitigating climate change to health care through an unprecedented collaboration between the British government, the World Health Organization (WHO), and Health Care Without Harm, underscoring the global recognition of the health care sector’s role in addressing climate change [6,7].

Following these international commitments, various countries have established national-level initiatives to address climate change in health care. The United Kingdom established a goal of achieving net-zero carbon emissions in health care through green nephrology and other initiatives [8]. In 2020, the United Kingdom launched its Greener National Health Service (NHS) initiative to establish the National Health Service as the world’s first net-zero emissions health care system, with this commitment formally embedded in the Health and Care Act passed on July 1, 2022, making the United Kingdom the first country to legislate net-zero emissions in health care [9]. Similarly, the United States has established the Office of Climate Change and Health Equity (OCCHE) as a cross-agency federal institution to facilitate the transition to net-zero carbon emissions in the health care sector [10]. These national efforts are complemented by global initiatives from the WHO [6] and nongovernmental organizations [7].

To understand the scope of this challenge, it is essential to define key concepts in sustainable health care. Net-zero carbon emissions refer to a dynamic equilibrium in which the volume of anthropogenic greenhouse gases emitted is offset by greenhouse gases removed. Hence, net-zero emissions do not entail the complete absence of emissions but rather the maximum reduction of anthropogenic greenhouse gases [6]. Carbon footprint is a quantifiable metric for comparing greenhouse gas emissions attributable to various activities, products, enterprises, or nations and is typically expressed in tons of CO_2_-equivalent (CO_2_-eq) per unit of measurement [6]. Overall, the health care industry’s contribution to global greenhouse gas emissions has been estimated at 4% to 6% [7], underscoring the importance of sustainable practices in medicine.

Building on these international and national commitments, countries worldwide continue to join this movement toward sustainable health care. On Earth Day 2021, Taiwan declared its commitment to net-zero emissions by 2050, subsequently announcing its 2050 net-zero emissions pathway on March 30, 2022, and officially passing its Climate Change Adaptation Act on February 15, 2023 [11]. In practical terms, green nephrology initiatives focus on reducing water and energy consumption, minimizing waste generation, promoting the use of renewable energy sources, and implementing efficient dialysis technologies. These comprehensive approaches not only reduce operational costs but also align kidney care with global sustainability objectives, demonstrating that environmental responsibility and quality patient care can be successfully integrated.

### Global Trends and Best Practices in Sustainable Kidney Care

Sustainable kidney care has gained increasing global attention in response to the dual challenge posed by climate change: its role in exacerbating kidney disease and the significant environmental footprint of nephrology services. Rising temperatures are shifting the epidemiology of kidney disease, with higher incidences of acute kidney injury (AKI), chronic kidney disease (CKD), and kidney stones—particularly among vulnerable populations with limited health care access [3,12,13]. Evidence shows that extreme heat days are associated with a 1.7% to 3.1% rise in emergency visits for kidney-related conditions [14]. Heat stress and dehydration are key contributors, promoting kidney stone formation and potentially increasing the risk of recurrent urinary tract infections [15]. Physiologically, high ambient temperatures lead to elevated core temperature, dehydration, and blood hyperosmolality, all of which stress the kidneys and increase heatstroke-related kidney injury risk. Climate change also facilitates the spread of vector- and water-borne infectious diseases, many of which can trigger AKI [13]. Extreme weather events like typhoons and floods may disrupt health care infrastructure, restrict access to dialysis and essential kidney care, and strain regional medical resources. Compounding these issues, low water intake from heat and water scarcity further raises the risk of dehydration-related kidney conditions, including heatstroke, kidney stones, and CKD [15,16]. Beyond the direct health impacts, climate change has also highlighted the substantial environmental burden of dialysis care itself. A seminal report in *Nature Reviews Nephrology* underscored the destabilizing effect of climate change on kidney disease care and revealed that in-center hemodialysis generates carbon emissions more than 7 times higher than those associated with routine inpatient or outpatient care in the United Kingdom, emphasizing the need for sustainable nephrology practices [2].

The carbon footprint associated with patients receiving dialysis has been extensively researched in several countries. The literature reveals that patients receiving dialysis generate an average of 3-10 tons of CO_2_-eq emissions annually (Table 1). Overall, the United Kingdom is a pioneer in terms of quantifying the carbon footprint of patients receiving nephrology care. In 2010, Connor et al [17] estimated that their 277 dialysis patients accounted for 65.4% of a renal center’s carbon emissions, totaling 1965 tons of CO_2_-eq, or 7.1 tons per person annually. A subsequent component analysis study in 2011 demonstrated that in-center patients undergoing hemodialysis receiving three 4-hour treatments weekly had an annual carbon footprint of 3.8 tons of CO_2_-eq, whereas patients receiving home hemodialysis undergoing 5.5 treatments weekly for 3 hours each had a footprint of 1.8 tons of CO_2_-eq emissions [18]. Chen et al [19] corroborated these findings, estimating a comparable carbon footprint of approximately 1.4 tons for Chinese patients receiving peritoneal dialysis at home.

**Table 1:** Summary of estimated CO_2_ emissions for patients on dialysis.

Author	Country	Year	Definition of outcome	Estimated CO_2_ emission (tons of CO_2_-eq)	Summary
Connor et al [17]	United Kingdom	2010	Per dialysis patient per year (dorset renal service)	7.1	For a regional renal service, providing hemodialysis and peritoneal dialysis to 277 patients produced 1965 tons of CO_2_-eq annually, equivalent to 7.1 tons of CO_2_-eq per dialysis patient per year.
Connor et al [18]	United Kingdom	2011	Per in-center hemodialysis patient per year	3.8	For in-center patients on hemodialysis, the carbon footprint was 3.8 tons of CO_2_-eq per patient per year.
Connor et al [18]	United Kingdom	2011	Per home hemodialysis patient per year (NxStage equipment)	1.8	For home hemodialysis patients, the carbon footprint was 1.8 tons of CO_2_-eq with NxStage short daily dialysis.
Lim et al [20]	Australia	2013	Per satellite hemodialysis patients per year	10.2	For dialysis patients in Australia, the annual per-patient carbon footprint of satellite hemodialysis was 10.2 tons of CO_2_-eq, with the largest contributors being pharmaceuticals (35.7%) and medical equipment (23.4%)​.
Chen et al [19]	China	2017	Per at-home peritoneal dialysis patient per year	1.4	For peritoneal dialysis patients in China, the annual per-patient carbon footprint was approximately 1.4 tons of CO_2_-eq, with packaging materials being the primary contributor (79.4%), followed by waste disposal (8.3%) and electricity (5%).
Mtioui et al [21]	Morocco	2021	Per in-center hemodialysis patient per year	5.1	For hemodialysis patients in Morocco, the annual per-patient carbon footprint was 5.11 tons of CO_2_-eq, with the primary contributors being electrical energy consumption (28%), equipment purchases and services (27%), and staff and patient travel (22%).
Sehgal et al [22]	United States	2022	Per hemodialysis treatment	0.0589	For hemodialysis patients in the United States, the emission volume per treatment was 58.9 kg CO_2_-eq, with the primary contributors being patient and staff transportation (28.3%), electricity (27.4%), and natural gas (15.2%).
Nagai et al [23]	Japan	2024	Per dialysis patient per year	3.9	For patients with CKD^a^ in Japan, those undergoing dialysis generated 3.9 tons of CO_2_-eq per year, whereas nondialysis patients produced 0.31 tons of CO_2_-eq per year. Among nondialysis patients, emissions increased with CKD severity: patients with stable CKD stage G2 generated 300 kg of CO_2_-eq (men) and 280 kg of CO_2_-eq (women), whereas those with CKD stages G3a and G3b experiencing a rapid decline in renal function produced up to 1440 kg of CO_2_-eq (men) and 1270 kg of CO_2_-eq (women).

^a^CKD: chronic kidney disease.

Other studies have reported varying carbon footprints associated with dialysis. Annual emissions per patient with dialysis have been estimated at 10.2 tons of CO_2_-eq in Australia [20], 5.1 tons of CO_2_-eq in Morocco [21], and 3.9 tons of CO_2_-eq in Japan [23], reflecting regional differences in health care infrastructure, energy sources, and treatment practices. Per-treatment emissions provide another perspective on the environmental effects of dialysis, with studies estimating 24.5 kg of CO_2_-eq per session in the United Kingdom [18], 65.1 kg of CO_2_-eq in Australia [20], 32.8 kg of CO_2_-eq in Morocco [21], and 58.9 kg of CO_2_-eq in the United States [22]. Despite these findings, direct comparisons across studies remain challenging because of differences between the contributors of emissions. A study conducted in Japan examined carbon footprint across different stages of CKD, illustrating how emissions increase with disease severity. The study demonstrated that patients undergoing dialysis generate 3.9 tons of CO_2_-eq annually, whereas patients not undergoing dialysis produce 0.31 tons of CO_2_-eq annually [23]. Additionally, among patients not undergoing dialysis, emissions increase as renal function declines. Patients with stable CKD stage G2 generate 300 kg of CO_2_-eq (men) and 280 kg of CO_2_-eq (women) annually, whereas those with CKD stages G3a and G3b, experiencing a rapid decline in renal function, produce up to 1440 kg of CO_2_-eq (men) and 1270 kg of CO_2_-eq (women).

Implementing sustainable practices in nephrology has substantial costs and social benefits across health care systems. The National Health Service in the United Kingdom projected potential savings of £1 billion (US $1.57 billion) from the adoption of green strategies in kidney units [2,24,25]. In 2013, England’s national clinical director for kidney care estimated that the successful replication of 20 green nephrology initiatives across kidney units may lead to annual savings of £7 million (US $11 million), a reduction of 11,000 tons in greenhouse gases, and the conservation of 470 million liters of water [24]. In France, the NephroCare network of Fresenius Medical Care reported substantial reductions in resource consumption across its dialysis units between 2005 and 2018. Their achievements included a 29.6% decrease in power consumption (from 23.1 to 16.26 kWh/session), a 52% reduction in water consumption (from 801 to 382 L/session), and an increase in annual dialysis sessions from 169,335 to 399,336 [26]. Care-related waste was also reduced from 1.8 to 1.1 kg per session owing to regular staff training and the implementation of a retro filtration system; these efforts mitigated the use of the equivalent of 102,440 tons of carbon dioxide. In Australia, Agar et al [27,28] conducted comprehensive assessments of water recycling in hemodialysis centers and the installation of solar energy systems at homes, demonstrating that investment costs of AU $5590 (US $4200) in hospitals and AU $1500-$2000 (US $1125-$1500) in homes resulted in savings of approximately 350 L of water per treatment. Additionally, an investment of AU $16,219 (US $12,160) led to a 91% reduction in grid power consumption, a 76.5% reduction in power costs, and a return on investment within 7-8 years. An Italian study examining 4 waste management policies in dialysis units reported that implementing a careful-optimal waste management approach required only an additional 1-2 min per dialysis session and saved between €45 and €52.5 million (US $50-58 million) in Italy alone, with estimated potential global cost savings of €3 billion (US $3.3 billion) [29]. The global experience in promoting green nephrology has consistently focused on electricity conservation, water efficiency, and waste recycling, with several countries reporting substantial health care savings from carbon reduction initiatives. Notably, the carbon reduction experiences in French dialysis centers have not compromised patient care, and the UK’s National Health Service has reported substantial savings from carbon reductions in nephrology. Therefore, although the adoption of sustainable practices in nephrology requires initial investment, the long-term savings in health care expenditures and the accompanying environmental benefits make this approach highly cost-effective [30].

### Proposed Model of a Net-Zero Emissions Kidney Care Center

Given that Taiwan has the world’s highest per-capita utilization of dialysis, implementing sustainable kidney care is an urgent concern [31]. According to the 2023 United States Renal Data System report, the incidence of dialysis in Taiwan in 2021 was 522 per million individuals, with a prevalence of 3839 per million individuals [31]. In Taiwan, CKD, including dialysis, is associated with a substantial health care expenditure of NT $53.3 billion (approximately US $1.8 billion), which represents a major economic burden. This burden is particularly concerning given that Taiwan’s National Health Insurance (NHI) system faces recurring financial challenges and structural deficits [32].

Taiwan’s health care sector is beginning to adopt measures promoting decarbonization and sustainability without compromising essential services. However, increasing pressure from disease burdens in aging societies and during pandemics may exacerbate carbon emissions if proactive measures are not implemented [33]. The country’s aging population has led to a steady increase in patients on dialysis, with older patients, particularly those older than 75 years of age, representing the fastest-growing demographic requiring dialysis [34]. Additionally, Taiwan’s health care system must navigate the dual pressures of maintaining universal health coverage while addressing environmental responsibilities, as the country has legally committed to achieving net-zero emissions by 2050 under its Climate Change Response Act [11]. The implementation of future carbon fees and “green inflation” risks poses additional financial challenges to health care institutions, making sustainable practices not just environmentally responsible but economically necessary.

Within this context, Taiwan’s exceptionally high prevalence of dialysis creates substantial potential for meaningful carbon footprint reductions through targeted nephrology initiatives. By adopting green nephrology practices and implementing preventive measures to reduce the number of patients on dialysis, Taiwan can achieve reductions in both health care expenditure and environmental impact. Taiwan’s health care sectors are beginning to adopt measures promoting decarbonization and sustainability. However, increasing pressure from disease burdens in aging societies and during pandemics may exacerbate carbon emissions. Therefore, establishing a net-zero emissions kidney care center is both an ecological conservation and a practical necessity that balances clinical excellence with environmental responsibility to address Taiwan’s unique health care challenges.

## Conceptual Framework and Objectives

In this study, we proposed a comprehensive conceptual framework for a kidney care center at Shuang Ho Hospital in Taipei, Taiwan. This framework responds to the global “Net Zero by 2050” action initiative proposed by the UN Framework Convention on Climate Change (UNFCCC COP26) [5] and aligns with Taiwan’s phased carbon reduction targets announced in 2022 [11]. This study aims to develop a Sustainable Development Goals (SDGs)–aligned framework [35] enabling health care institutions to implement low-carbon transformation while maintaining quality kidney care. Expected outcomes include: (1) a practical implementation guide for Shuang Ho Hospital’s net-zero transition, (2) a replicable reference framework for other health care institutions, and (3) demonstration of how clinical care can align with environmental sustainability goals.

To ensure both theoretical foundation and practical feasibility, we first conducted comprehensive research synthesis through literature review to examine evidence on CKD and environmental impacts of dialysis treatment, policy benchmarking against international standards such as the United Kingdom’s Greener NHS initiative [9] and the US OCCHE [10], and case synthesis of domestic and international hospital experiences in energy conservation, circular economy, and renewable energy implementation. We then grounded the theoretical foundation in the GREEN-K initiative’s three strategic dimensions: education advocacy and collaborative promotion, procurement mechanisms with infrastructure innovation, and sustainable clinical pathways [36]. Additionally, we adopted the systematic thinking approach proposed by Luyckx et al [37], which informed the cross-sector integration strategy, emphasizing that kidney care requires coordinated governance across climate, energy, social policy, and education sectors.

**Figure 1:**
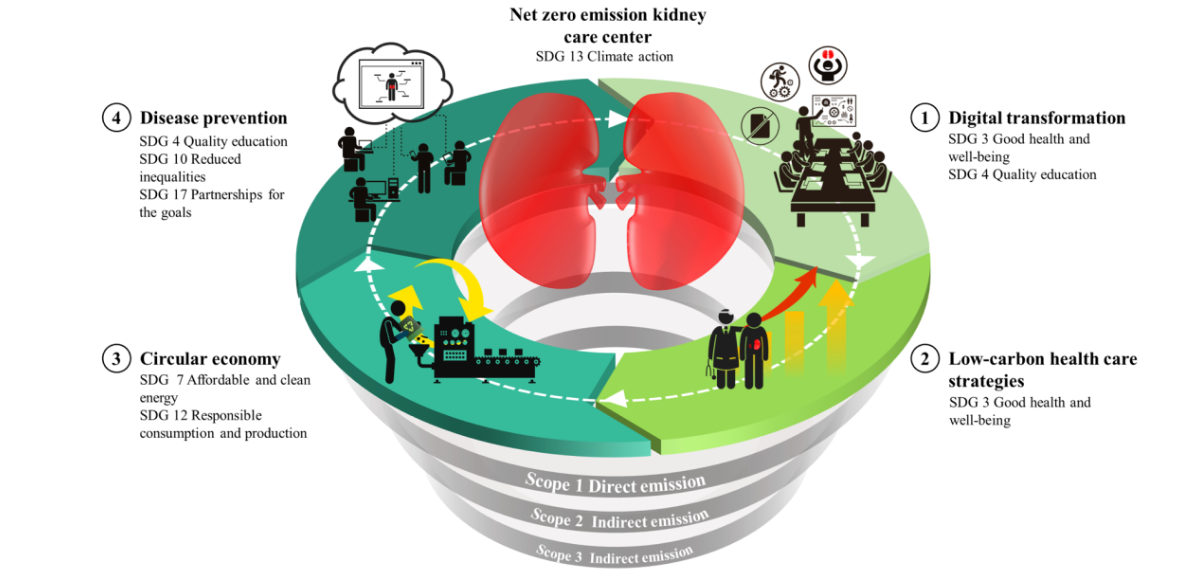
Conceptual framework of the proposed net-zero emissions kidney care center, aligned with the United Nations’ SDGs. This figure illustrates a multilayered framework for sustainable kidney care, with a kidney-shaped core representing SDG 13 (Climate Action) and the goal of net-zero emissions. Four strategic dimensions extend outward: (1) digital transformation (SDGs 3 and 4) for improved health care and education, (2) low-carbon health care strategies (SDG 3) for sustainable practices, (3) circular economy (SDGs 7 and 12) for renewable energy and responsible resource use, and (4) disease prevention (SDGs 4, 10, and 17) for equitable care and global collaboration. The 3 concentric layers at the bottom represent scope 1 (direct), scope 2 (indirect), and scope 3 (indirect) emissions, offering a structured approach to emission tracking and reduction. SDG: Sustainable Development Goal.

This framework is structured in concentric layers, each representing a strategic dimension with corresponding objectives (Figure 1). Based on a comprehensive review of the literature examining the interactions between all 17 SDGs and kidney health, which revealed high correlations between CKD and goals related to climate action, educational resources, responsible consumption, and health equity, we therefore positioned SDG 13 (Climate Action) as the central axis [37,38]. Each dimension strategically corresponds to specific SDGs to address these interconnected challenges. The first dimension focuses on digital transformation, based on SDG 3 (Good Health and Well-being) and SDG 4 (Quality Education). This dimension involves the implementation of advanced health information systems, telemedicine, and continuing medical education programs to enhance patient care and professional development. The second and third dimensions address low-carbon health care and circular economy principles, based on the SDG 3 (Good Health and Well-being), SDG 7 (Affordable and Clean Energy), and SDG 12 (Responsible Consumption and Production). These dimensions involve strategies such as enhanced kidney transplant promotion and care procedures to reduce mortality from noncommunicable diseases, together with renewable energy implementation, energy-efficient systems, sustainable procurement, waste reduction, and overall resource efficiency in health care settings. The last dimension involves domestic and international disease prevention, based on SDG 4 (Quality Education), SDG 10 (Reduced Inequalities), and SDG 17 (Partnerships for the Goals). This comprehensive approach focuses on equitable access to kidney care services and preventive measures across diverse populations in Taiwan. It requires collaboration between national and international partners to address kidney health challenges and promote knowledge delivery, research partnerships, and capacity-enhancing initiatives. This dimension emphasizes bridging local and global efforts in kidney care, fostering innovation, and promoting health equity across different geographical and socioeconomic settings.

The expected outcomes include serving as a practical foundation for Shuang Ho Hospital’s implementation and providing a reference framework for other health care institutions in planning climate action strategies, thereby strengthening the health care system’s resilience and competitiveness under climate change and international sustainability standards. This comprehensive approach emphasizes cross-cutting themes such as innovation, interdisciplinarity, and integration of social and environmental factors, representing a globally oriented, locally implementable, and scalable sustainable governance model committed to enhancing the resilience and sustainability of kidney health care systems under climate risk challenges.

## Four Dimensions of the Model

### Overview

This section outlines the four key dimensions of the model, detailing the specific strategies implemented under each and their anticipated impacts on kidney care and sustainability. To further clarify how these strategies contribute to the achievement of the SDGs, Table 2 presents a mapping of each dimension’s initiatives to relevant SDGs, highlighting the alignment between the proposed actions and the broader global sustainability agenda.

**Table 2:** Four dimensions of the model and the corresponding SDGs^a^, specific targets, and practical actions.

Dimensions	Corresponding SDGs	The targets	Practical actions
Proposed overall model	SDG 13 Climate Action	13.2 Integrate climate change measures into policies and planning 13.B Promote mechanisms to raise capacity for planning and management	Proposed model of a net-zero emissions kidney care center
Digital transformation	SDG 3 Good Health and Well-being SDG 4 Quality Education	3.4 Reduce mortality from noncommunicable diseases and promote mental health 3.8 Achieve universal health coverage 4.7 Education for sustainable development and global citizenship	Comprehensive kidney health digital system Innovative service model for acute kidney injury
Low-carbon health care	SDG 3 Good Health and Well-being	3.4 Reduce mortality from noncommunicable diseases and promote mental health	Enhanced kidney transplant promotion and care procedures.
Circular economy	SDG 7 Affordable and Clean Energy SDG 12 Responsible Consumption and Production	7.2 Increase the global percentage of renewable energy 7.3 Double the improvement in energy efficiency 12.5 Substantially reduce waste generation	Renewable energy Energy efficiency systems. Recycling and reuse of hemodialysis waste and RO^b^ wastewater. Standardized dialysis procedures for resource optimization.
Disease prevention	SDG 4 Quality Education SDG 10 Reduced Inequalities SDG 17 Partnerships for the Goals	4.7 Education for sustainable development and global citizenship 10.2 Promote universal social, economic, and political inclusion 17.16 Enhance the global partnership for sustainable development	Telehealth and behavioral interventions. Domestic and international partnerships: partnered with Taiwan Kidney Foundation to enhance kidney health education and access; support the development of Marshall Islands kidney care system International collaboration: Marshall Islands kidney care system development.

^a^SDG: Sustainable Development Goal.

^b^RO: reverse osmosis.

### Digital Transformation

#### Strategic Context

Climate change represents challenges that demand proactive approaches in health care, in which digital transformation plays a crucial role in strengthening medical teams’ resilience. By adopting digital information systems, kidney care centers can streamline their patient information management, reduce paper use, and improve resource efficiency. These advancements reduce the carbon footprint of traditional practices while enhancing the quality and effectiveness of medical services.

#### Comprehensive Kidney Health Digital System

**Figure 2:**
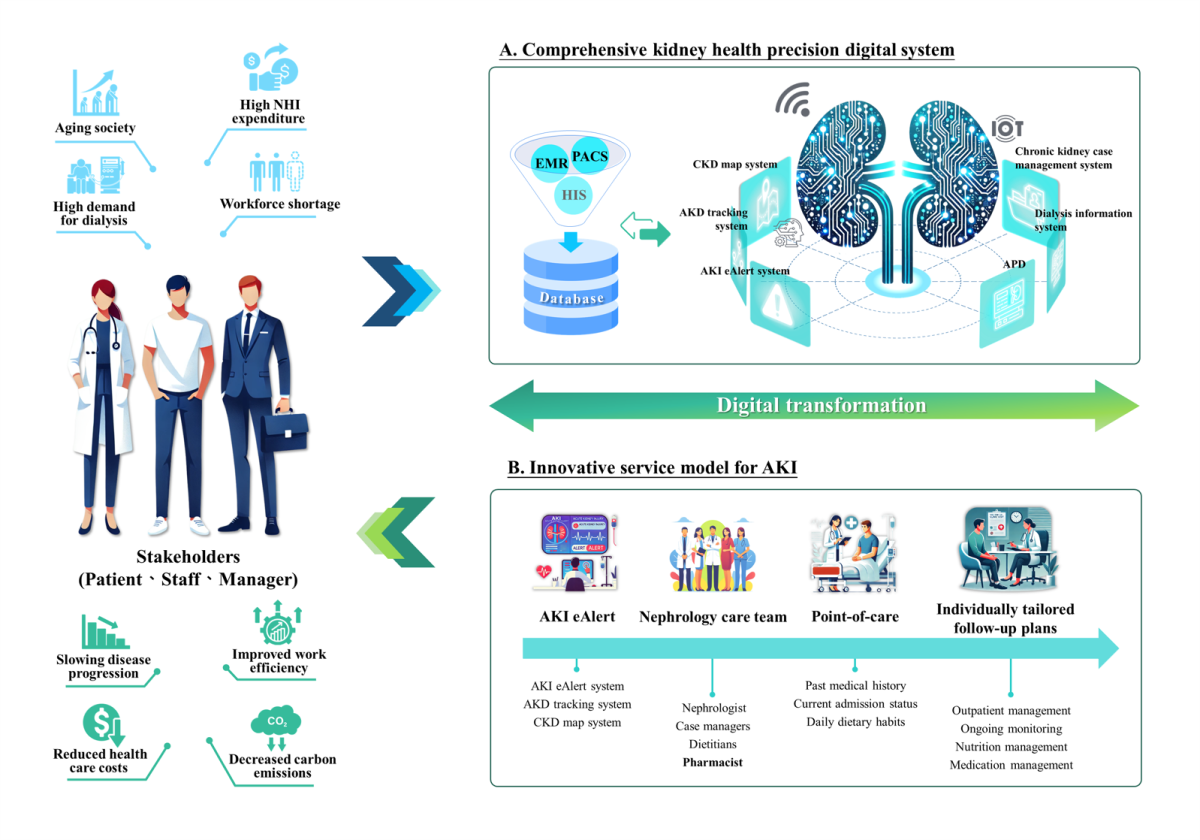
Digital transformation in kidney care: a comprehensive system and service model. The figure illustrates the integration of digital health solutions and innovative service models for kidney care. (A) Comprehensive kidney health digital system incorporating an EMR, a PACS, and an HIS, connected to specialized modules, including a CKD map system, an AKI tracking system, and a dialysis management platform. (B) Innovative AKI service model featuring 4 key components: AKI eAlert system, nephrology care team collaboration, point-of-care services, and individualized follow-up plans. AKI: acute kidney injury; AKD: acute kidney disease; APD: automated peritoneal dialysis; CKD: chronic kidney disease. EMR: electronic medical record; HIS: hospital information system; PACS: picture archiving and communication system.

A key component of this transformation is the development of a comprehensive kidney health precision digital system for the management of acute and CKD (Figure 2). Designed in collaboration with IT experts and integrated into the hospital information system, it enables health care providers to rapidly review patient status and laboratory reports, tracking disease progression over time and enabling timely interventions. For instance, when the estimated glomerular filtration rate falls below 45 mL/min/1.73m^2^, automated alerts prompt nutritional and pharmaceutical assessments to slow disease progression and reduce long-term medical burdens.

This digital system also incorporates a dialysis information system to facilitate electronic medical order entry, nursing record, and patient handover processes, thereby facilitating clinical operations and communication. The Internet of Things integration enables real-time data transfer from dialysis machines and electronic scales, improving workflow. Additionally, a built-in continuous venovenous hemofiltration prescription calculator automates the reading of hematocrit level, total dehydration volume, and filtration fraction, thereby replacing traditional manual techniques. This innovation has reduced the rates of mortality among patients with critical illness and minimized the waste of artificial kidney materials. Another key feature is a cloud-based management platform for automated peritoneal dialysis telemedicine, which remotely monitors dialysis and records key parameters like blood pressure, weight, and treatment volume. This approach enables timely interventions, improves care efficiency, and eliminates reliance on paper records. Preliminary data showed that digital case management enabled the recovery of kidney function in 104 patients with early-stage CKD in 2024, helping reduce an estimated 41.6 tons of CO_2_-eq, based on literature-derived emission values by disease stage [23]. In addition, digital documentation systems replaced approximately 49,900 sheets of paper annually, corresponding to a further reduction of around 0.9 tons of CO_2_-eq.

#### Innovative Service Model for AKI

AKI leads to severe complications if not promptly identified and managed. The proposed service model integrates multiple early identification strategies to enhance patient outcomes, emphasizing a structured in-hospital strategy that prioritizes the early detection of AKI through rigorous monitoring and patient education (Figure 2). By detecting AKI early, timely interventions can be initiated. Point-of-care education and transitional care assessments ensure patients receive personalized follow-up plans. Given that 18% to 27% of AKI cases are drug-related, pharmacists play a crucial role in this model. Integrating pharmacists into nephrology care teams, alongside nephrologists, case managers, and dietitians, can facilitate comprehensive medication management, reducing the risk of drug-induced AKI. In addition to its functionalities, the proposed model incorporates digital tools to identify high-risk medications and increase care precision. Research indicates that the severity of kidney disease is correlated with carbon emissions [23]. This model not only improves patient outcomes but also supports sustainability by preventing disease progression and reducing unnecessary treatments. In 2008, over 2 million people had CKD in Taiwan, yet only 3.5% are aware of their condition [39], leading to delayed diagnosis and increased AKI risk. Studies indicate that AKI episodes significantly raise the likelihood of developing CKD, even if serum creatinine levels return to baseline [40]. To address these challenges, our team developed a kidney function early warning system in 2020 [41], integrating inpatient monitoring, early detection measures, and multidisciplinary care to comprehensively track disease trajectories and enhance health literacy and outcomes. This model aligns with multiple SDGs by improving disease prognosis, increasing efficiency, reducing unnecessary treatments, and minimizing waste. According to 2024 data, application of the AKI care model led to improvement or stabilization in kidney function for 11 patients, resulting in an estimated reduction of 11.4 tons of CO_2_-eq, as calculated from changes in disease stage–specific emission factors [23].

### Low-Carbon Health Care Strategies

#### Strategic Context

In Taiwan, more than 90,000 patients undergo dialysis, and approximately 12,000 new cases are added each year. This treatment produces carbon emissions 10 to 20 times higher than those generated by typical nondialysis inpatient or outpatient care. To reduce environmental impact and improve treatment efficiency, our team developed low-carbon health care strategies aimed at decreasing dialysis dependence by promoting kidney transplantation and optimizing care procedures.

#### Enhanced Kidney Transplant Promotion and Care Procedures

Kidney transplantation, the preferred option for patients with end-stage kidney disease or those seeking to discontinue long-term dialysis, is actively promoted through public awareness campaigns and shared decision-making strategies. A satellite care model has been introduced to expand access to living donor transplantation. To address the shortage of organ donors, frontline medical staff are trained to identify and approach potential donors, supported by the Organ Donation and Transplantation Registration Center, which collaborates with the Ministry of Health and Welfare and medical institutions to refine donor identification guidelines and facilitate quicker matching of donors and recipients. Each successful kidney transplant can reduce carbon emissions by an estimated 2.0 to 3.6 tons of CO_2_-eq annually, compared to continued dialysis treatment [23].

Taiwan’s organ transplantation system operates under comprehensive regulatory oversight established by the Human Organ Transplantation Act, which ensures ethical practices and privacy protection throughout the donation process. All organ donations must be conducted on a noncommercial basis with strict confidentiality maintained for both donor and recipient information. For living organ donation, donors must be adults providing written consent under free will, with additional written certification from their nearest relatives. Living donor transplantation is generally limited to recipients within the fifth degree of kinship, while unrelated donors are matched through the national Organ Procurement Registry and Allocation System to ensure medical suitability and allocation fairness. Within our health care institution, the medical team confirms donor voluntariness, mental competence, and physical eligibility while prioritizing donor safety throughout the organ procurement process. All procedures require Institutional Review Board (IRB) approval to ensure ethical oversight, and comprehensive medical records are maintained for transparency.

In addition, the decision aid tool, Is a Kidney Transplant Right for Me?, provides patients with evidence-based information, while transplant coordinators engage with long-term patients on dialysis to facilitate evaluations and registrations. Posttransplant care is strengthened through an integrated team of physicians, surgeons, transplant coordinators, and pharmacists, who conduct comprehensive preoperative and postoperative assessments. To standardize care and reduce organ rejection risks, the Adult Kidney Transplant Medication Care Guide has been implemented through structured training sessions for clinical staff.

### Circular Economy

#### Strategic Context

A circular economy model has been introduced to manage the environmental impact of dialysis treatment. Because of the substantial resource consumption and waste generation associated with hemodialysis—the production of 2.5-8 kg of medical waste, the consumption of 12-19.6 kWh of electricity, and the use of 500 L of reverse osmosis (RO) water per session—this initiative focuses on recycling and reusing resources to reduce carbon emissions and enhance sustainability [2].

#### Recycling and Reuse of Hemodialysis Waste and RO Wastewater

A comprehensive waste management strategy has been implemented to address the environmental impact of dialysis treatments. Approximately 4 tons of RO wastewater are generated daily, which is treated through specialized collection and recycling systems and repurposed for nonportable uses such as toilet flushing, reducing overall water consumption. Dialysis-related waste, including disposable catheters, filters, gloves, gowns, and lab coats that encounter patient blood, is carefully sorted using colored bags and clear labeling to ensure proper handling and recycling. Staff members receive training to adhere to strict waste management protocols.

Internally, plastic medicine bottles made of low- or high-density polyethylene are recycled into environmentally friendly cleaning bags through industry collaboration, involving processes such as breakdown, washing, pelletizing, and bag production. These 100% recycled bags received environmental certification in March 2024. Externally, waste materials such as polyvinyl chloride from artificial kidneys and blood tubing are processed into plastic pellets for manufacturing, while pharmaceutical glass vials are repurposed into construction materials like bricks, asphalt, and tiles to reduce carbon emissions and disposal costs. Altogether, the implementation of these circular initiatives—including RO water recycling and hemodialysis waste repurposing—achieved an estimated reduction of over 384 tons of CO_2_-eq in 2024. By integrating these waste management and circular economy practices, the facility has effectively minimized its environmental footprint and set a model for sustainable health care operations.

#### Renewable Energy and Energy Efficiency Systems

Given the energy-intensive nature of dialysis treatment, implementing renewable energy systems is crucial for achieving net-zero emissions in kidney care centers [2,42,43]. International experience demonstrates feasibility, with Australia’s solar-assisted hemodialysis program showing that solar arrays can successfully support dialysis operations in a cost-effective manner [27,28], while NephroCare achieved a 30% reduction in energy consumption per dialysis session from 23.1 to 16.26 kWh through equipment upgrades and system optimization [26].

Our hospital has implemented a comprehensive renewable energy and energy efficiency strategy. In 2024, a 300.26 kW solar photovoltaic system was installed, with an estimated annual generation capacity of 294,126 kWh, resulting in approximately 133.4 tons of CO_2_-eq reduction annually. The system operates under a hybrid model combining self-consumption and grid feed-in, with approximately 14,591 kWh directly supplying internal hospital operations. While rooftop space and upfront investment pose practical constraints, these were addressed through phased implementation and participation in national renewable energy incentive programs. In addition, energy efficiency improvements complement our renewable infrastructure through integrated systems. Complete light-emitting diode lighting upgrades, combined with motion sensor systems, generate annual savings of approximately 530,000 kWh. Five regenerative braking elevator systems contribute an additional 140,000 kWh in recovered energy annually. Advanced heat pump systems using waste heat account for 500,000 kWh in electricity savings each year, while smart chilled water control technologies further reduce consumption by approximately 1.31 million kWh annually.

The effectiveness of these initiatives is evidenced by ISO 50001 energy management certification and measurable performance improvements. In 2024, the hospital’s energy intensity reached 221 kWh/m^2^, outperforming the national average for medical centers in Taiwan (224 kWh/m^2^) and exceeding international benchmarks. Each kWh generated NT $358 (US $11) in medical revenue in 2024, representing a 19% improvement since 2021, indicating enhanced energy efficiency that outpaces electricity consumption growth.

#### Standardized Dialysis Procedures for Resource Optimization

The AEIOU (acidosis, electrolyte imbalance, intoxication, overload, and uremia) mnemonic is a widely recognized clinical tool used to remember common indications for initiating dialysis, particularly in patients with AKI. AEIOU represents five critical clinical scenarios: acidosis (severe metabolic acidosis), electrolyte imbalance (particularly life-threatening hyperkalemia), intoxication (from dialyzable toxins), overload (fluid overload unresponsive to conservative management), and uremia (symptomatic uremia with complications). This standardized mnemonic approach helps clinicians systematically evaluate the appropriate timing for initiating or discontinuing dialysis, thereby optimizing resource utilization while ensuring patient safety and reducing unnecessary treatment. When a patient exhibits one or more of the AEIOU indicators and fails to respond to other interventions—as in cases of uncontrolled hyperkalemia or severe acidosis—dialysis is recommended to prevent further complications. The proposed protocol outlines criteria for discontinuing dialysis, ensuring that treatment is only administered when absolutely necessary. For example, dialysis may be discontinued if a patient’s renal function substantially recovers, as indicated by a urine output exceeding 400 mL within 24 hours without the use of diuretics or 1000 mL with diuretic support.

### Preventive Medicine

#### Strategic Context

Net-zero emissions kidney care centers prioritize disease prevention to reduce carbon emissions while improving patient and public health outcomes. Digital platforms provide kidney health education, focusing on preventing hypertension and diabetes, the primary risk factors for kidney disease. By minimizing in-person consultations, these platforms lower travel-related emissions while expanding access to health information. Additionally, telemedicine services enable continuous remote CKD management, reducing energy and resource use while ensuring early detection and preventing disease progression.

#### Telehealth and Behavioral Interventions

Expanded telemedicine services provide comprehensive remote management of chronic kidney conditions through integrated clinical monitoring and behavioral interventions. These services enable continuous patient assessment while reducing hospital visits, energy consumption, and resource use. Patients receive personalized guidance for lifestyle modifications, including balanced nutrition, regular physical activity, and smoking cessation. This approach maintains high care standards while reducing reliance on resource-intensive treatments and their environmental impact.

#### Addressing Health Inequities and Social Determinants

While Taiwan’s universal health care system ensures equitable access to preventive kidney care services, our framework actively addresses health inequities through targeted initiatives for vulnerable populations. Domestically, our center collaborates with the Kidney Disease Prevention Foundation, a nongovernmental organization dedicated to improving kidney health education and health care access in underserved communities. This partnership specifically targets remote townships and mountainous tribal areas across Taiwan through comprehensive community outreach programs, including kidney disease screening activities, elementary school health education, accessible health education materials, low-protein cooking education, exercise promotion, and smoking cessation support.

Internationally, we address global health inequities through partnerships with resource-limited regions. Our collaboration with the Marshall Islands Ministry of Health exemplifies this initiative, developing sustainable kidney care systems through clinical capacity building, telemedicine consultations, web-based medical knowledge exchange platforms, advanced degree programs for local health care personnel, and comprehensive dialysis center planning. This collaboration provides immediate clinical support and long-term capacity building to establish autonomous and sustainable health care operations. Additionally, partnerships with environmental organizations integrate sustainability into kidney care through renewable energy use, sustainable waste management, and green initiatives, ensuring high-quality care while minimizing environmental impact.

## Governance Structure and Maintenance Strategies

### Financial and Implementation Considerations

The successful implementation of a net-zero emissions kidney care center requires substantial initial investments in renewable energy systems, digital infrastructure, and waste recycling facilities. Key capital expenditures include solar energy installations, advanced digital health platforms, Internet of Things-integrated dialysis equipment, and comprehensive waste processing systems. While upfront costs represent a significant financial commitment, long-term operational savings through reduced energy consumption, improved resource efficiency, and minimized waste disposal costs demonstrate a favorable return on investment [44].

The financing strategy combines multiple funding sources to ensure sustainability and scalability. Primary funding is secured through government health care innovation grants, reflecting Taiwan’s net-zero emissions commitment by 2050. Additionally, corporate partnerships provide supplementary funding as part of their corporate social responsibility initiatives, while public-private partnerships facilitate knowledge transfer and technology sharing.

### Patient and Staff Engagement in Sustainable Health Care Practices

To ensure stakeholder buy-in for sustainable transformation, the implementation of our net-zero emissions kidney care center involves active engagement of both health care professionals and patients. To foster staff commitment, structured surveys assess health care workers’ knowledge and attitudes toward climate change and carbon reduction, providing a foundation for targeted training programs that encourage low-carbon clinical behaviors and support broader institutional transformation.

Staff engagement includes regular educational sessions on waste management protocols, energy-saving practices, and sustainable medical procedures. These training programs integrate environmental consciousness into daily clinical routines while maintaining high patient care standards. Through continuous professional development, staff members develop competencies in resource optimization, waste segregation, and energy-efficient equipment operation.

For patient engagement, we have implemented comprehensive health education programs that emphasize the connection between environmental sustainability and personal health outcomes. Patients receive guidance on reducing their carbon footprint through sustainable lifestyle choices and health care–seeking behaviors. Our center provides shuttle bus services to reduce individual transportation emissions, enhancing both emission reduction and health care accessibility through reliable transportation alternatives.

### Institutional Governance Framework and Committee Structure

The governance framework adopts a committee-based structure that integrates Environmental, Social, Governance principles regarding organizational governance, environmental management, and social responsibility [45,46]. This approach incorporates insights from the GREEN-K initiative [36], the United Kingdom’s Greener NHS [8], US OCCHE policies [10], Taiwan’s Climate Change Response Act [11], and systems thinking methodology [47] to develop a health care–specific and practically feasible sustainability structure [9,10,36].

Our hospital has established a comprehensive sustainable development committee under the leadership of the hospital director. This 15-member committee, which comprises all deputy directors and key departmental heads, convenes semiannually to guide sustainability efforts. The governance framework includes 6 specialized Environmental, Social, Governance promotion groups: sustainable governance, risk response, medical excellence, workplace happiness, sustainable environment, and social participation. Each group is led by a senior executive and a director-level executive secretary to ensure accountability. These groups are responsible for developing organizational philosophies, implementing projects, and optimizing outcomes while disseminating sustainable practices and fostering collaboration throughout the organization. The sustainable development committee integrates projects from all groups and monitors progress for cohesive implementation. Within the nephrology team, responsibilities are allocated based on expertise, with resource management promoting leadership, vigilance, cooperation, and communication. Progress is evaluated during monthly administrative quality meetings, supported by educational initiatives including journal clubs, lectures, case discussions, and interdisciplinary conferences to maintain cutting-edge kidney care knowledge.

Beyond institutional goals, the hospital engages with national and international partners to develop innovative medical services through collaborative research and to mitigate climate-change-related disease outcomes. This comprehensive strategy promotes sustainable kidney care and addresses local and global health challenges through environmentally conscious practices.

## Ethical Considerations

The study was approved by the Joint IRB of Taipei Medical University (TMU-JIRB Approval No. N202404142). Given that the research involved only deidentified secondary data, the requirement for informed consent was waived by the IRB. All study procedures were performed in accordance with institutional and international ethical guidelines and regulations.

## Conclusions and Recommendations

With the world actively pursuing the net-zero emission goals of 2050, achieving a sustainable transformation in the field of health care has become an urgent topic. Our net-zero emissions kidney care center model responds to the challenges of climate change through its multidimensional framework integrating digital technology, low-carbon strategies, circular economy, and preventive medicine. This model emphasizes enhancing efficiency through digital transformation, reducing dialysis demand through organ transplantation, transitioning to renewable energy sources, implementing waste recycling, and strengthening preventive care. These initiatives can reduce carbon emissions and enhance health care quality and resource utilization. In Taiwan, where the NHI system offers universal coverage, integrating sustainability into chronic disease care is crucial to ensure long-term financial and ecological resilience. This model demonstrates how existing infrastructures—such as NHI-supported CKD management and hospital IT systems—can be aligned with climate goals.

To support widespread adoption of sustainable kidney care practices, several policy recommendations are proposed. Short-term initiatives should include targeted financial incentives for health care institutions adopting green technologies and streamlined regulatory approval processes for sustainable medical innovations. Medium-term objectives focus on extending the net-zero kidney care model to neighboring community dialysis centers, creating an integrated low-carbon kidney care network that maximizes regional environmental benefits while maintaining care accessibility. Long-term goals involve scaling successful experiences nationally and internationally, positioning Taiwan as a global leader in sustainable health care innovation.

This model represents a fundamental shift in health care delivery, indicating that environmental sustainability and clinical excellence need not be mutually exclusive and can be mutually reinforced. As health care systems worldwide confront the challenges of providing quality care and minimizing environmental influence, this framework offers a practical roadmap for sustainable transformation that can be adapted and implemented across diverse health care contexts.

## Abbreviations

AEIOU: acidosis, electrolyte imbalance, intoxication, overload, uremia
AKI: acute kidney injury
CKD: chronic kidney disease
IRB: Institutional Review Board
NHI: National Health Insurance
NHS: National Health Service
OCCHE: Office of Climate Change and Health Equity
RO: reverse osmosis
SDG: Sustainable Development Goals
WHO: World Health Organization
